# Premanifest Huntington’s disease: Examination of oculomotor abnormalities in clinical practice

**DOI:** 10.1371/journal.pone.0193866

**Published:** 2018-03-01

**Authors:** Jessica Y. Winder, Raymund A. C. Roos

**Affiliations:** Department of Neurology, Leiden University Medical Center, Leiden, The Netherlands; University of Pennsylvania Perelman School of Medicine, UNITED STATES

## Abstract

**Introduction:**

Different oculomotor abnormalities have been reported to occur in premanifest Huntington’s disease. The aim of this study is to investigate which oculomotor items of the Unified Huntington’s Disease Rating Scale (UHDRS) are affected in premanifest individuals compared to healthy controls, and if CAG repeat length and age are correlated with oculomotor abnormalities in premanifest Huntington’s disease gene carriers.

**Methods:**

We compared baseline data of 70 premanifest individuals and 27 controls who participated in the Enroll-HD study at the Leiden University Medical Center, the Netherlands. Premanifest gene carriers were divided in individuals near to disease onset and individuals far from disease onset.

**Results:**

Using a logistic regression model, only horizontal ocular pursuit of the six oculomotor items of the UHDRS was significantly more frequently affected in premanifest individuals close to disease onset compared to controls (*p* = 0.044, OR 13.100). Age was significantly higher in premanifest individuals with affected horizontal ocular pursuit (*p* = 0.016, OR 1.115) and with affected vertical ocular pursuit (*p* = 0.030, OR 1.065) compared to premanifest individuals without ocular pursuit deficits.

**Conclusions:**

Our results suggest that horizontal ocular pursuit is the only affected oculomotor item of the UHDRS in premanifest individuals and could be used to assess early clinical signs of Huntington’s disease. Saccade initiation and saccade velocity do not seem useful for detecting differences between premanifest individuals and controls.

## Introduction

Huntington’s disease (HD) is an autosomal dominant neurodegenerative disorder characterized by progressive motor, cognitive and psychiatric symptoms. It is caused by an expanded cytosine-adenine-guanine (CAG) trinucleotide repeat in the Huntingtin gene on chromosome 4 [1]. The mean age at onset is between 30 and 50 years, with a range of 2 to 85 years [2].

Many studies have focused on the identification of potential biomarkers in premanifest HD. The standard clinical assessment tool for HD is the Unified Huntington’s Disease Rating Scale (UHDRS) [3]. The PREDICT-HD study showed that premanifest individuals closer to estimated age of disease onset had worse scores in the chorea, the bradykinesia, and the oculomotor domain of the UHDRS than individuals further from estimated diagnosis [4].

Several cross-sectional studies have shown that eye movements are impaired in an early stage of HD, often long before other symptoms become clinically relevant. These studies with eye-tracking equipment have found abnormal antisaccade and memory guided tasks, variability of latency, and error rates [5–8]. However, eye-tracking equipment is not easily accessible in a clinical setting. Instead, the oculomotor items of the UHDRS are much easier to use in clinical practice.

The aim of our study is to determine if the oculomotor items of the UHDRS show relevant abnormalities in premanifest HD. We also want to examine if a higher CAG expansion is associated with more oculomotor abnormalities in premanifest HD individuals compared to lower CAG expansions, considering CAG repeat length is inversely correlated with age of disease onset [9,10]. Additionally, we aim to examine the relationship of age on oculomotor deficits in premanifest HD individuals, since oculomotor abnormalities also occur under the influence of ageing in healthy people [11,12].

## Materials and methods

Baseline data of subjects participating in the Enroll-HD study at the Leiden University Medical Center (LUMC), the Netherlands, were included in this study (S1 File). Enroll-HD is an observational, prospective, international, multi-center study without experimental treatment. The Medical Ethics Committee of the LUMC approved the study (P13.167) and written informed consent was obtained from all participants. Assessments performed in this study include the examination of motor functioning using the UHDRS-Total Motor Score (TMS). Testing conditions were uniform across all subjects, and according to the instructions of the UHDRS-TMS teaching film [13]. The oculomotor assessments were performed before the other motor assessments. The UHDRS-TMS was performed by four different raters. The raters were not blinded to the status of the participants. The six oculomotor items of the UHDRS-TMS are horizontal and vertical ocular pursuit, horizontal and vertical saccade initiation, and horizontal and vertical saccade velocity (Table 1). They can all be rated from 0 ‘normal’ to 4 ‘cannot perform’. Overall, higher scores indicate more severe motor impairment.

**Table 1:** The oculomotor items of the Unified Huntington’s Disease Rating Scale.

Ocular pursuit (horizontal and vertical)	0 = complete (normal)
	1 = jerky movement
	2 = interrupted pursuit/full range
	3 = incomplete range
	4 = cannot pursue
Saccade initiation (horizontal and vertical)	0 = normal
	1 = increased latency only
	2 = suppressible blinks or head movements to initiate
	3 = unsuppressible head movements
	4 = cannot initiate saccades
Saccade velocity (horizontal and vertical)	0 = normal
	1 = mild slowing
	2 = moderate slowing
	3 = severely slow, full range
	4 = incomplete range

A total of 326 participants visited the neurology department of the LUMC for a baseline visit of Enroll-HD between October 2014 and September 2016. Manifest HD patients (*n* = 220) and participants with an unknown genotype (*n* = 9) were excluded. Seventy premanifest HD individuals and 27 controls (genotype negative individuals (*n* = 14), family controls (*n* = 12) and community controls (*n* = 1)) were included. Premanifest gene carriers were defined as having a total motor score of 5 or less on the UHDRS-TMS. Premanifest HD gene carriers were divided in premanifest individuals near to disease onset and premanifest individuals far from disease onset by the group median for expected years to HD onset (13.7 years). Expected years to onset was calculated for each individual using the formula described by Langbehn et al. [14], which is based on CAG repeat length and age at visit. Genotype negative individuals were potentially at risk for HD, but tested negative. Family controls were non-related family members of HD gene carriers (mainly husbands and wives). The community control volunteered and did not visit the neurology department before.

Statistical analysis was performed using IBM Statistical Package for the Social Sciences (SPSS) version 23. Demographics were calculated using independent sample t-tests and Chi-square tests. A linear regression analysis of the UHDRS-TMS as a function of age was performed among controls. Comparisons between premanifest individuals and controls for the six oculomotor items were performed using a logistic regression model, adjusting for age and gender. The UHDRS total oculomotor score is the sum of the separate oculomotor items and comparisons for this score between premanifest individuals and controls were assessed using a linear regression model, controlling for age and gender. The relationships between oculomotor abnormalities and CAG repeat length and age were calculated using a logistic regression model, controlling for gender. The oculomotor items served as dependent variables and CAG repeat length and age as independent variables. A *p*-value of <0.05 was considered statistically significant.

## Results

Demographic data are shown in Table 2. Premanifest HD gene carriers were significantly younger (*p* < 0.001) than controls and had a lower UHDRS-TMS (*p* = 0.005). In the control group, UHDRS-TMS was not related to age (*p* = 0.271). CAG repeat length was available for a minority of the controls, because it was not determined in non-related family controls and community controls who were not at risk for HD. Premanifest individuals closer to disease onset were older (*p* = 0.002), had a higher CAG expansion (*p* < 0.001), and a higher UHDRS-TMS (*p* = 0.015) compared to premanifest HD gene carriers further from disease onset.

**Table 2:** Demographic data divided by gene status and expected years to HD onset.

	PreHD (*n* = 70)	PreHD near (*n* = 35)	PreHD far (*n* = 35)	Controls (*n* = 27)	Controls versus preHD		PreHD near versus preHD far	
					MD/OR (95% CI)	*p*-value	MD/OR (95% CI)	*p*-value
Age, years	39.6 (±10.4)	43.4 (±9.9)	35.8 (±9.6)	50.9 (±13.5)	11.340 (6.238–16.442)	**<0.001**	7.571 (2.920–12.223)	**0.002**
Gender, male	23 (32.9%)	14 (40.0%)	9 (25.7%)	14 (51.9%)	0.454 (0.184–1.123)	0.084	1.926 (0.697–5.319)	0.203
CAG repeat length	42.1 (±2.4)	43.4 (±2.1)	40.8 (±2.0)	NA	NA	NA	2.571 (1.604–3.539)	**<0.001**
UHDRS-TMS	1.5 (±1.7)	2.0 (±1.8)	1.0 (±1.5)	2.7 (±2.0)	1.152 (0.357–1.948)	**0.005**	0.971 (0.193–1.750)	**0.015**

Data are mean (±standard deviation) for age, CAG repeat length, and UHDRS-TMS, and number (%) for gender. *p*-values were calculated using independent sample t-tests for age, CAG repeat length, and UHDRS-TMS, and Chi-square tests for gender. MD are given for age, CAG repeat length, and UHDRS-TMS; OR are given for gender.

HD, Huntington’s disease; PreHD, premanifest individuals; preHD near, premanifest individuals near to HD onset; preHD far, premanifest individuals far from HD onset; MD, mean difference; OR, odds ratio; CI, confidence interval; NA, not applicable; UHDRS-TMS Unified Huntington’s Disease Rating Scale-Total Motor Score.

All subjects scored 0 ‘normal’ or 1 ‘mild abnormality’ on the six oculomotor items of the UHDRS, and were therefore classified as ‘not affected’ or ‘affected’ respectively. Of the six oculomotor items, vertical ocular pursuit was the most frequently affected item in premanifest individuals (32.9%) as well as in controls (22.2%) (Table 3). Horizontal saccade velocity was not affected once in the entire group. When comparing all premanifest gene carriers to controls no statistically significant differences were found for the total UHDRS oculomotor score, or any of the six separate oculomotor items. However, when premanifest HD individuals near to disease onset were compared with healthy controls a significant difference was seen for horizontal ocular pursuit (*p* = 0.044, OR 13.100), with more premanifest individuals affected than controls. A similar trend was observed for vertical ocular pursuit (*p* = 0.075, OR 2.967). Horizontal and vertical saccade initiation, and horizontal and vertical saccade velocity did not show any differences between the groups.

**Table 3:** Relationship of premanifest individuals and controls with the oculomotor items of the UHDRS.

	PreHD (*n* = 70)	PreHD near (*n* = 35)	PreHD far (*n* = 35)	Controls (*n* = 27)	PreHD versus controls		PreHD near versus controls	
					OR/B (95% CI)	*p*-value	OR/B (95% CI)	*p*-value
Ocular pursuit horizontal, affected	8 (11.4%)	6 (17.1%)	2 (5.7%)	1 (3.7%)	11.325 (0.927–138.333)	0.057	13.100 (1.072–160.165)	**0.004**
Ocular pursuit vertical, affected	23 (32.9%)	15 (42.9%)	8 (22.9%)	6 (22.2%)	2.333 (0.719–7.570)	0.158	2.967 (0.896–9.823)	0.075
Saccade initiation horizontal, affected	3 (4.3%)	2 (5.7%)	1 (2.9%)	1 (3.7%)	4.019 (0.276–58.608)	0.309	3.991 (0.258–61.692)	0.322
Saccade initiation vertical, affected	8 (11.4%)	5 (14.3%)	3 (8.6%)	5 (18.5%)	0.918 (0.222–3.791)	0.906	1.003 (0.230–4.372)	0.996
Saccade velocity horizontal, affected	0 (0.0%)	0 (0.0%)	0 (0.0%)	0 (0.0%)	NA	NA	NA	NA
Saccade velocity vertical, affected	1 (1.4%)	0 (0.0%)	1 (2.9%)	3 (11.1%)	0.128 (0.009–1.733)	0.122	<0.001	0.998
UHDRS total oculomotor score	0.61 (±0.997)	0.80 (±1.052)	0.43 (±0.917)	0.59 (±0.844)	-0.243 (-0.712–0.225)	0.305	-0.175 (-0.418–0.067)	0.154

Data are number (%) for the separate oculomotor items and mean (±standard deviation) for the UHDRS total oculomotor score. *p*-values were calculated using a logistic regression model for the separate oculomotor items and a linear regression model for the UHDRS total oculomotor score. Both models were controlled for age and gender. OR are given for the separate oculomotor items; B are given for the UHDRS total oculomotor score.

HD, Huntington’s disease; UHDRS, Unified Huntington’s Disease Rating Scale; PreHD, premanifest individuals; PreHD near, premanifest individuals near to HD onset; PreHD far, premanifest individuals far from HD onset; OR, odds ratio; B, beta; NA, not applicable.

The relationship between the oculomotor items of the UHDRS and CAG repeat length and age was examined in premanifest individuals, controlling for gender. CAG repeat length was not related to horizontal and vertical ocular pursuit (Table 4). Age was significantly higher in the affected horizontal ocular pursuit group (*p* = 0.016, OR 1.115) and the affected vertical ocular pursuit group (*p* = 0.030, OR 1.065) compared to premanifest individuals without ocular pursuit deficits.

**Table 4:** Relationship of CAG repeat length and age in premanifest individuals (*n* = 70) with horizontal and vertical ocular pursuit.

	Ocular pursuit horizontal				Ocular pursuit vertical			
	Affected (*n* = 8)	Not affected (*n* = 62)	OR (95% CI)	*p*-value	Affected (*n* = 23)	Not affected (*n* = 47)	OR (95% CI)	*p*-value
CAG repeat length	42.1 (±2.1)	42.1 (±2.5)	1.227 (0.787–1.913)	0.366	42.5 (±3.0)	41.9 (±2.0)	1.229 (0.956–1.581)	0.107
Age, years	48.0 (±11.1)	38.5 (±9.9)	1.115 (1.021–1.219)	**0.016**	42.8 (±12.0)	38.0 (±9.3)	1.065 (1.006–1.127)	**0.030**

Data are mean (±standard deviation) for CAG repeat length and age. *p*-values were calculated using a logistic regression model, adjusting for gender.

OR, odds ratio; CI, confidence interval.

## Discussion

In this cross-sectional study we showed that only horizontal ocular pursuit of the UHDRS oculomotor domain is affected in premanifest individuals near to HD onset compared to healthy controls. This is not the case when all premanifest HD gene carriers are compared with controls. This suggests that horizontal ocular pursuit is the only affected item of the six oculomotor items of the UHDRS in premanifest individuals. Vertical ocular pursuit showed a tendency towards the same.

Our demographic data showed that premanifest individuals near to HD onset were older, had a higher CAG expansion, and a higher UHDRS-TMS compared to premanifest HD gene carriers far from HD onset. These findings were expected, since age and CAG repeat length were used to calculate the expected years to HD onset according to the Langbehn formula [14]. The higher UHDRS-TMS found in controls compared to premanifest individuals is probably due to the higher age of the participants in this group, which has been reported before [15]. However, a linear regression analysis of the UHDRS-TMS as a function of age among controls did not show this correlation.

To our knowledge no study has been performed investigating the separate items of the oculomotor domain of the UHDRS in premanifest HD individuals and controls. Other cross-sectional reports using clinical oculomotor assessments only showed that premanifest individuals performed significantly worse compared to controls on the total score of the oculomotor domain of the UHDRS [4], and on the overall oculomotor function, saccade velocity, and optokinetic nystagmus of the Quantified Neurologic Examination [16]. Our study did not find a difference for the total UHDRS oculomotor score between controls and premanifest individuals. This could be caused by a different definition used for premanifest HD individuals, which caused lower total oculomotor scores on the UHDRS-TMS in our study. Biglan et al. [4] used a diagnostic confidence level to identify premanifest individuals. As a result, individuals with an UHDRS-TMS higher than 5 were often defined as premanifest, because participants were only defined as manifest when the examiner was more than 98 percent confident the participant had signs of HD. Accordingly, patients with a relatively high UHDRS-TMS were categorized as premanifest rather than manifest, while those same patients would have been categorized as manifest in our study. Our study’s definition of premanifest HD, therefore, selects for lower UHDRS-TMS scores and, accordingly, the UHDRS total oculomotor scores were also lower in premanifest HD gene carriers in our study.

Differences found between controls and premanifest HD gene carriers are important, because they increase knowledge about early disease progression, and possibly indicate the first clinical signs present in premanifest HD. Since eye-tracking equipment is not practical in everyday clinical practice, it is relevant to know if the oculomotor items of the UHDRS are useful to detect early HD signs in patients. Our results suggest that horizontal ocular pursuit is the only affected oculomotor item in premanifest individuals and could be used to assess early clinical signs of HD in individuals who are at risk for developing HD. Vertical ocular pursuit was not significantly different between premanifest individuals close to disease onset and controls, but a trend was seen for this item as well. Horizontal and vertical saccade initiation, and horizontal and vertical saccade velocity did not show any differences between premanifest individuals and controls or between premanifest individuals close to disease onset and controls. Therefore these items of the UHDRS do not seem to contribute in detecting early disease signs. The fact that we did not find significant differences does not necessarily mean that they are not present, but might show that these items are not sensitive enough to defect deficits. Siesling et al. [17] also questioned the importance of the eye movements, because omitting saccade initiation and saccade velocity from the UHDRS-TMS only led to a small loss in correlation between the other items.

In contrast, other studies did find differences between premanifest individuals and controls for saccade initiation and saccade velocity [5–8]. However, these studies used eye-tracking equipment. They reported oculomotor abnormalities between premanifest HD gene carriers and controls, which consisted of more complex antisaccade and memory guided tasks, variability of latency, and error rates. However, in the TRACK-HD study [18], antisaccade error rates in controls did not differ from those in premanifest individuals, only from those in premanifest individuals closer to predicted HD onset. Eye-tracking equipment did not show significant differences for horizontal and vertical pursuit tracking between premanifest individuals and controls [5]. Only one study compared results from eye-tracking equipment with clinical ratings of the UHDRS oculomotor section. Saccade initiation of the UHDRS was correlated with the average latency of saccades measured with the eye-tracking system. The correlation between the saccade velocity of the UHDRS and the measured velocity was not significant [6].

In the second part of our study we examined the relationship between CAG repeat length and oculomotor abnormalities, and age and oculomotor abnormalities in premanifest HD individuals. Because saccade initiation and saccade velocity did not show differences between premanifest individuals and controls, we only examined ocular pursuit. We did not find a relationship between CAG repeat length and ocular pursuit. Age, however, was related to both horizontal and vertical ocular pursuit. This suggests that a clinician should be more aware of the possibility of affected ocular pursuit in older premanifest HD gene carriers. Especially since older individuals are more likely to be closer to HD onset.

A limitation of our study is the relatively small sample size and therefore the small number of participants who had oculomotor abnormalities. Secondly, the examiners were not blinded to the status of the participants. As a result, equivocal findings may have been graded as abnormal in premanifest HD gene carriers and normal in controls, thereby biasing results against the null hypothesis. Clinical examination of the oculomotor items of the UHDRS has its limitations due to difficulty in distinguishing between subtle possible pathology and normal eye movements, and due to interrater reliability. Previous studies have shown that the interrater reliability of the total UHDRS motor score is high, however they did not report the interrater reliability of the UHDRS oculomotor domain [3,19]. Additionally, eye movement abnormalities can have a nonspecific nature as well. Furthermore, we have tested multiple hypothesis without accounting for multiple comparisons. We did not correct for multiple comparisons because of the relatively small sample size and the likely presence of correlations between the different items. Accordingly, significant findings may be due to chance alone. If we want to decide whether or not the UHDRS oculomotor domain is as good as eye-tracking equipment to determine oculomotor abnormalities, these two assessments should be assessed together in an observational, prospective study.

## Conclusions

In conclusion, we found that significantly more premanifest HD individuals near to disease onset had affected horizontal ocular pursuit compared to controls. This suggests that horizontal ocular pursuit is the only affected oculomotor item in premanifest individuals and could be used to assess early clinical signs of HD. Saccade initiation and saccade velocity do not seem useful for detecting differences between premanifest individuals and healthy controls. Therefore, when assessing individuals at risk for HD, these items of the UHDRS-TMS might be omitted. Our results also showed that higher age is related to horizontal and vertical ocular pursuit deficits in premanifest individuals. CAG repeat length was not related to oculomotor abnormalities.

## Supporting information

S1 File All data underlying the findings in this manuscript. (XLSX)

## References

[1] Huntington’s Disease Collaborative Research Group. A novel gene containing a trinucleotide that is expanded and unstable on Huntington’s disease chromosomes. Cell. 1993;72:971–983. 8458085 10.1016/0092-8674(93)90585-e

[2] Roos RAC. Huntington’s disease: a clinical review. Orphanet J Rare Dis. 2010;5:40 doi: 10.1186/1750-1172-5-40 21171977 10.1186/1750-1172-5-40 PMC3022767

[3] Huntington Study Group. Unified Huntington’s Disease Rating Scale: reliability and consistency. Mov Disord. 1996;11:136–142. doi: 10.1002/mds.870110204 8684382 10.1002/mds.870110204

[4] Biglan KM, Ross CA, Langbehn DR, Aylward EH, Stout JC, Queller S, et al Motor abnormalities in premanifest persons with Huntington’s disease: the PREDICT-HD study. Mov Disord. 2009;24:1763–1772. doi: 10.1002/mds.22601 19562761 10.1002/mds.22601 PMC3048804

[5] Blekher TM, Yee RD, Kirkwood SC, Hake AM, Stout JC, Weaver MR, et al Oculomotor control in asymptomatic and recently diagnosed individuals with the genetic marker for Huntington’s disease. Vision Res. 2004;44:2729–2736. doi: 10.1016/j.visres.2004.06.006 15358067 10.1016/j.visres.2004.06.006

[6] Blekher T, Johnson SA, Marshall J, White K, Hui S, Weaver M, et al Saccades in presymptomatic and early stages of Huntington disease. Neurology. 2006;67:394–399. doi: 10.1212/01.wnl.0000227890.87398.c1 16855205 10.1212/01.wnl.0000227890.87398.c1

[7] Antoniades CA, Altham PME, Mason SL, Barker RA, Carpenter R. Saccadometry: a new tool for evaluating presymptomatic Huntington patients. Neuroreport. 2007;18:1133–1136. doi: 10.1097/WNR.0b013e32821c560d 17589313 10.1097/WNR.0b013e32821c560d

[8] Hicks SL, Robert MPA, Golding CVP, Tabrizi SJ, Kennard C. Oculomotor deficits indicate the progression of Huntington’s disease. Prog Brain Res. 2008;171:555–558. doi: 10.1016/S0079-6123(08)00678-X 18718352 10.1016/S0079-6123(08)00678-X

[9] Duyao M, Ambrose C, Myers R, Novelletto A, Persichetti F, Frontali M, et al Trinucleotide repeat length instability and age of onset in Huntington’s disease. Nat Genet. 1993;4:387–392. doi: 10.1038/ng0893-387 8401587 10.1038/ng0893-387

[10] Stine OC, Pleasant N, Franz ML, Abbott MH, Folstein SE, Ross CA. Correlation between the onset age of Huntington’s disease and length of the trinucleotide repeat in IT-15. Hum Mol Genet. 1993;2:1547–1549. 8268907 10.1093/hmg/2.10.1547

[11] Moschner C, Baloh RW. Age-related changes in visual tracking. J Gerontol. 1994;49:235–238. 10.1093/geronj/49.5.m235 8056943

[12] Seferlis F, Chimona TS, Papadakis CE, Bizakis J, Triaridis S, Skoulakis C. Age related changes in ocular motor testing in healthy subjects. J Vestib Res. 2015;25:57–66. doi: 10.3233/VES-150548 26410670 10.3233/VES-150548

[13] Reilmann R, Roos R, Rosser A, Grimbergen Y, Kraus P, Craufurd D, et al A teaching film, video library and online certification for the Unified Huntington’s Disease Rating Scale Total Motor Score. Akt Neurol. 2009; 36: 474.

[14] Langbehn DR, Brinkman RR, Falush D, Paulsen JS, Hayden MR. A new model for prediction of the age of onset and penetrance for Huntington’s disease based on CAG length. Clin Genet. 2004;65:267–277. doi: 10.1111/j.1399-0004.2004.00241.x 15025718 10.1111/j.1399-0004.2004.00241.x

[15] Cubo E, Ramos-Arroyo MA, Martinez-Horta S, Martinez-Descalls A, Calvo S, Gil-Polo C. Clinical manifestations of intermediate allele carriers in Huntington disease. Neurology. 2016;87:1–8.

[16] Kirkwood SC, Siemers E, Bond C, Conneally PM, Christian JC, Foroud T. Confirmation of subtle motor changes among presymptomatic carriers of the Huntington disease gene. Arch Neurol. 2000;57:1040–1044. 10891987 10.1001/archneur.57.7.1040

[17] Siesling S, Zwinderman AH, Van Vugt JPP, Kieburtz K, Roos RAC. A shortened version of the motor section of the Unified Huntington’s Disease Rating Scale. Mov Disord. 1997;12:229–234. doi: 10.1002/mds.870120214 9087982 10.1002/mds.870120214

[18] Tabrizi SJ, Langbehn DR, Leavitt BR, Roos RAC, Durr A, Craufurd D, et al Biological and clinical manifestations of Huntington’s disease in the longitudinal TRACK-HD study: cross-sectional analysis of baseline data. Lancet Neurol. 2009;8:791–801. doi: 10.1016/S1474-4422(09)70170-X 19646924 10.1016/S1474-4422(09)70170-X PMC3725974

[19] Youssov K, Dolbeau G, Maison P, Boissé M-F, Cleret de Langavant L, Roos RAC, et al The Unified Huntington’s Disease Rating Scale For Advanced Patients: validation and follow-up study. Mov Disord. 2013;28:1995–2001. doi: 10.1002/mds.25678 24123464 10.1002/mds.25678

